# On the Perspirable Fluids

**Published:** 1800-07

**Authors:** 


### *On the Perspirable Fluids*; *by* Dr. Mitchill.

From the American Journals we present our Readers with the following very ingenious "*Speculations concerning the perspirable Fluids of Human Bodies.* In a Letter from Dr. Mitchill to Dr. Duncan.

"Sir,

"In reply to your last letter, I have to remark, that, of the fluids afforded by the human body, few have been more talked of, though, perhaps, none to less advantage, than those exhalations or effusions from its surface, denominated *perspiration* or *sweat.* The reason alleged wherefore so little has been experimentally done upon these fluids or humours, is, that they cannot be obtained in sufficient quantity; their volatility being such as to prevent the collection of either of them, to such an amount, that much certain information can be obtained about their peculiar natures and qualities.

"This is, indeed, in some degree, the case; and, without doubt, the difficulty of procuring, conveniently and frequently, parcels of the matter secreted from the skin, has retarded the progress of inquiry on this point. While, therefore, the fluids secreted in the liver, pancreas, kidneys, stomach, mouth, and almost every other part of the human frame, have been examined with great care and exactness, experimenters are yet very much behind-hand in their knowledge of the cuticular discharge.

"It is not my intention to strike the balance between the proportions of fluids secreted by the kidneys and the skin, on the one part, nor to adjust the difference between the exhalation by arteries or glands, and the inhalation by lymphatics or absorbents, on the other. My object is, to examine, by such means as, it appears to me, we are possessed of, what substances they are which compose the cuticular secretion in the time of health, what alterations it undergoes by accumulation and confinement in certain cases, and how pestilential fluids and fomites may be produced thereby.

"It will not, therefore, be necessary to give an anatomical description of the skin. All that will be offered will be capable of being comprehended without going into details upon that subject. Almost all that you need recollect is, that the *true skin* is furnished with arteries, veins, glands, excretory ducts, and lymphatics, and that it is covered over by a porous, semi-transparent sort of sheath, called epidermis, or cuti

CLE, which is *inſenſible*, *bibulous*, and *exceedingly apt to grow dirty*; and this to a conſiderable degree without the conſciouſneſs of the wearer.

" A quantity of oil, ſecreted into the cells of the adipoſe membrane, underlays the true ſkin in moſt parts of the body; and the veſſels and glands concerned in this function combine alſo a portion of the phlogiſton (hydrogen) and carbon of the blood, into a ſebaceous or greaſy compoſition, to anoint and ſoften the cuticle. A portion of this fatty matter, in the time of health, is ſpread over the whole ſurface, though in greater quantity in ſome parts, and at ſometimes, than at others. (Liquor oleoſus—inflammabilis—cutem perungit. *Haller*, 5 Element. Phyſiolog. § 20.) The palms of the hands, the inſides of the fingers, and the ſoles of the feet, are remarked for having the leaſt of it.

" This oily fluid, with which the external ſurface of the body is ſmeared, was found, in the early ſtages of ſociety, while yet dreſs was ſimple, and few or no clothes were worn, to be too copious in ſome conſtitutions, and to collect in greater quantity than the purpoſes of comfort required. When this happened, from its adheſive nature, particles of duſt, and other things floating in the air, would readily ſtick to it, and occaſion ſome degree of unpleaſant ſenſation. To remove this unpleaſantneſs, and the cauſe of it, inſtinct or accident led to the practice of *bathing*, or of purification with *water*; which being not capable of removing completely the foul and greaſy collection, was afterwards quickened in its activity by *alkalies* and *soaps*, ſubſtances which experience proved to be very efficacious in ridding the ſkin of its incumbrance. It ſometimes happened, too, that the oily liniment of the ſkin was too ſparingly effuſed, or too quickly removed, and did not impart to the cuticle (which, it will be remembered, has neither nerves nor ſenſibility) that pliancy and ſuppleneſs which is eſſential to pleaſant and perfect feeling. The epidermis was then prone to become horny, to crack, and to induce diſagreeable or painful ſenſation. To relieve this inconvenience, the cuſtom of applying *grease* or *oil* over the ſurface of the body ſeems to have been adopted by mankind likewiſe at a very early period; and in this way the deficiency of the matter of ſecretion was ſupplied by art.

" The uſe of water, and of alkaline lotions, ſometimes rendered more active by being warmed, and the employment of oil and ſoft ointments, now and then aided by *manual friction*, ſeem to have obtained very extenſively, from the mere comfortable effects they wrought, without any reaſoning or theory whatever. Sometimes a perſon employed the *watery* application tion only, sometimes the *oily*, and then again he made use of *both*, in rapid succession.

"But this adipose secretion is by no means a *pure oil.* According to circumstances, it is occasionally blended with *phosphoric*, *sulphuric*, *septic*, and a surplusage of *carbonic* matter.

"And it is further altered, as far as its nature will permit, by the constant transmission of that AQUEOUS FLUID, the INSENSIBLE PERSPIRATION, through the cuticle, and by the drops of SWEAT into which it is sometimes condensed; both formed by a coalition of a portion of the phlogiston (hydrogen) of the blood, with a parcel of its oxygen, and this liquid serving as a menstruum for certain other substances. The exceedingly great quantity of perspired matter incessantly passing off from every exhalant pore of the skin, leaves behind, as it evaporates, some of its more fixed, saline, and feculent parts, along with, or upon, the greasy covering of the cuticle. A portion of carbon, phosphorus, and septon, with, sometimes, an overplus of oxygen, seem to accompany this effusion of the skin, and, in some quantity, to remain behind, after the water has evaporated.

"It was remarked before, that the cuticle was insensible, bibulous, and, of course, exceedingly apt to grow dirty. How can it be otherwise? The cuticle has neither nerves nor blood-vessels, and there is little or no reason to believe that it possesses organization. It is therefore, in strictness, less a part of the living body, than a kind of tight shirt, or close-fetting tunic, drawn over the whole surface of the body. This shirt or tunic of cuticle may be considered as a foreign wrapper, or piece of natural clothing; and, like every other foreign thing which enwraps the body, is liable to become charged with such substances as are excreted from the TRUE SKIN within it. The foulness of this NATURAL SHIRT is a frequent occurrence. Almost every body lets it get dirty. When the nastiness is very considerable, the poisonous or stimulant matter entangled there, irritates or inflames the organ of feeling, with which it lies in contact, and produces various itchings, pimples, eruptions, blotches, tetters, sores, &c. being a PRINCIPAL CAUSE of the distempers of the skin. To a correct mind, *a dirty cuticle will appear to be a dirty shirt.* If the former could be stripped off, and cleansed in the wash-tub, like the latter, there might possibly be a convenience in it. This is, however, of small moment, since we know the cuticle can be well cleansed without being taken off.

"The modern use of white linen and cotton, as articles of raiment, enables us to judge, with tolerable accuracy, what material it is, which shirts, made of those kinds of cloth, and worn next the cuticle, receive from the PERSPIRATION and SWEAT. What the garments imbibe muft have paffed firft through the exhalant arteries or excretory ducts, then undergone a further change while remaining in and about the CUTICLE or FIRST SHIRT, and afterwards have been tranfmitted, by rubbing or wiping, to the linen fibres or cotton filaments OF THE SECOND. The animal matter which befouls the OUTER SHIRT is a good indication of that which adheres TO THE INNER. For, as clean linen and cotton become foiled very foon by lying in contact with any part of the human body, what they receive muft be principally derived from the cuticle which they touch and abfterge.

"The uncleannefs of SHIRTS, DRAWERS, and STOCKINGS, may, therefore, be deemed to be matter wiped from the CUTICLE, and derived to the cuticle from the veffels and ducts of the fubjacent TRUE SKIN. An examination, then, of the facts relative to clothing grown foul by long wearing next the cuticle, may be confidered as virtually an examination of the cuticle itfelf, and of the excreted matters which ftick to it.

"I. One general property of all thefe matters, and of clothes tainted by them, is, that alkaline falts and foaps will feize, neutralize, and extract them; the garments being thereby rendered wholefome and clean. An unqueftionable effect of fuch applications is to remove the *greafy* or *unctuous* matter communicated to them from the cuticle. But this is not all—The *watery* as well as the *oily* part of the perfpirable matter contains the BASES OF ACIDS. A portion of oxygen combining with this fat material, imparts to it more or lefs of rancidity. Another portion, uniting to carbon, forms carbonic acid, and this, volatilized by heat, is converted into carbonic acid gas. Another portion of the acidifying principle, uniting to the phofphorus, turns it to phofphoric acid: while the fepton (azot), if it happens to combine with caloric alone, flies off in the form of azotic air; or, if it makes a junction with oxygen, conftitutes, with it, SEPTIC ACID. Oils, being capable of uniting with the ftronger acids, efpecially with the feptic, and probably, alfo, with the phofphoric and carbonic, there can be little doubt that thefe acids, or fome of them, or fome part of fome of them, join with the adipofe matter into a fort of ACID SOAP, on the cuticle and fhirt. This acidifying procefs, whereby carbon, phofphorus, and fepton, unite to oxygen, takes place, NOT BEFORE SECRETION, but AFTER the perfpired matter is depofited on the cuticle, or lodged in the fhirt. The formation of acids, in this cafe, is not the immediate effect of vital movement at all, but refults from chemical attraction going on among the particles of the fluids,

AFTER

AFTER EXCRETION, as in other inanimate matter; neither the cuticle, nor the fluids which besmear and bedew it, having any living energy to controul or modify the laws of chemical action. Hence proceeds that SOUR SMELL from clothes in which sailors and labouring men have sweated profusely, which, though obvious to the senses, and familiar to the wearers and to washer-women, remains unknown to hardly any body but philosophers.

[ To be continued. ]

